# Hydrogen Sulfide Regulates Cardiovascular Function by Influencing the Excitability of Subfornical Organ Neurons

**DOI:** 10.1371/journal.pone.0105772

**Published:** 2014-08-21

**Authors:** Markus Kuksis, Pauline M. Smith, Alastair V. Ferguson

**Affiliations:** Queen’s University, Department of Biomedical and Molecular Sciences, Kingston, Ontario, Canada; University of Toronto, Canada

## Abstract

Hydrogen sulfide (H_2_S), a gasotransmitter endogenously found in the central nervous system, has recently been suggested to act as a signalling molecule in the brain having beneficial effects on cardiovascular function. This study was thus undertaken to investigate the effect of NaHS (an H_2_S donor) in the subfornical organ (SFO), a central nervous system site important to blood pressure regulation. We used male Sprague-Dawley rats for both *in vivo* and *in vitro* experiments. We first used RT-PCR to confirm our previous microarray analyses showing that mRNAs for the enzymes required to produce H_2_S are expressed in the SFO. We then used microinjection techniques to investigate the physiological effects of NaHS in SFO, and found that NaHS microinjection (5 nmol) significantly increased blood pressure (mean AUC = 853.5±105.7 mmHg*s, n = 5). Further, we used patch-clamp electrophysiology and found that 97.8% (88 of 90) of neurons depolarized in response to NaHS. This response was found to be concentration dependent with an EC_50_ of 35.6 µM. Coupled with the depolarized membrane potential, we observed an overall increase in neuronal excitability using an analysis of rheobase and action potential firing patterns. This study has provided the first evidence of NaHS and thus H_2_S actions and their cellular correlates in SFO, implicating this brain area as a site where H_2_S may act to control blood pressure.

## Introduction

Hydrogen sulfide (H_2_S), while classically thought of as a poisonous substance, has recently been classified as the third gasotransmitter and plays an important role in cardiovascular function through the regulation of blood pressure [1]–[5]. H_2_S was first discovered to play important roles in the central nervous system through modulation of long term potentiation in the hippocampus [6]. Endogenously, H_2_S is produced in different parts of the body through a variety of pathways involving four different enzymes. Cystathionine β-synthase (CBS) is highly expressed in the brain and produces H_2_S from cysteine [6]. Cystathionine γ-lyase (CSE) also produces H_2_S from cysteine, but is mainly expressed in the liver, pancreas, aorta, ileum, portal vein, and uterus [7]–[10]. In the mitochondria, 3-mercaptopyruvate sulfur transferase (3MST) works in tandem with cysteine aminotransferase (CAT) to produce H_2_S via the breakdown of cysteine and α-ketoglutarate [11]. H_2_S is also obtained through dietary means by ingestion of polysulfide containing foods, which are then converted to H_2_S in the body by red blood cells [12]. While difficult to measure, endogenous concentrations of H_2_S have been estimated to be between 10 nM and 160 µM, with the lower of these values believed to be representative of total tissue levels, while the higher values of approximately 100 µM have been postulated to represent those found in local active pools through which H_2_S actually exerts its physiological actions [13]. While a variety of effects of H_2_S have been identified in the body, cellular production and storage is still not fully understood. H_2_S is stored in the brain as either acid-labile sulfur (mitochondria) or bound sulfur (cytoplasm) [14]. Neurons (under physiological concentrations of glutathione and cysteine in a slightly alkaline environment) and astrocytes (under high K^+^ conditions) release H_2_S from bound sulfur, while H_2_S is released from acid-labile sulfur under acidic conditions [15]. This free H_2_S is then able to contribute to physiological processes in the brain.

At these physiological concentrations, H_2_S has important roles in the nervous and cardiovascular systems. In the nervous system, H_2_S acts as a neuromodulator by influencing neuronal excitability through K^+^ channels [16]–[19] and Ca^2+^ channels [20], by inducing Ca^2+^ waves in astrocytes [21], and by facilitating long term potentiation in the hippocampus [6]. H_2_S exerts neuroprotectant effects by protecting against oxidative stress [22], [23], and has also been reported to both reduce tissue damage and enhance functional recovery following spinal cord injury [24]. In the cardiovascular system, H_2_S has important effects on blood pressure regulation both peripherally and centrally. It plays a role in both vasodilation and vasoconstriction in the superior mesenteric artery, an effect found to be concentration-dependent with higher concentrations (1 mM) causing vasodilation and lower concentrations (10 µM) causing vasoconstriction [2]. CSE knockout rats have increased blood pressure [3] and when H_2_S is administered via chronic intraperitoneal injections to hypertensive rats, their blood pressure is decreased [1], suggesting an important role for H_2_S in the control of blood pressure. Centrally, administration of H_2_S via intracerebroventricular (ICV) injection into the left ventricle induced transient hypotension followed by extreme hypertension [4]. Further, microinjection of H_2_S into the rostral ventrolateral medulla (RVLM) elicited overall decreases in blood pressure [18]. The role of H_2_S in the forebrain regulation of blood pressure has also recently been recognized, with Gan and colleagues showing cardiovascular effects of H_2_S on blood pressure and heart rate following microinjection of H_2_S into the paraventricular nucleus (PVN) [5]. We have also reported cellular correlates of such action using patch-clamp recordings to show a depolarizing effect of H_2_S on the majority of PVN neurons [19]. Although the above studies all support clear roles for H_2_S in cardiovascular regulation, the precise roles of H_2_S in the global regulation of blood pressure are likely very complex and tissue specific, and thus have yet to be clearly elucidated.

The subfornical organ (SFO) and other circumventricular organs (CVOs) lack a normal blood-brain barrier. They play important roles in sensing circulating signals from the periphery and sending such information to control centres behind the blood-brain barrier responsible for the regulation of autonomic responses [25]. The SFO has been shown to play critical roles in the control of blood pressure by integrating signals from a wide range of circulating neuropeptides including vasopressin [26], [27], orexin [28], apelin [29], angiotensin II [30], [31], atrial natriuretic peptide [32], and leptin [33]. It has also recently been shown that both NAPDH oxidases and superoxide both play a critical role in the SFO regarding angiotensin II induced hypertension, with site specific knockdown in SFO reducing hypertension induced by angiotensin II infusion [34], [35]. Our recent transcriptomic analysis of the SFO demonstrated expression of the mRNA for all four enzymes responsible for H_2_S synthesis in this CVO [36], observations which suggest possible actions of H_2_S in the SFO. As such, the present study was undertaken to investigate the potential roles of this gasotransmitter in the SFO. We used sodium hydrosulfide hydrate (NaHS), a fast H_2_S donor, in both microinjection and patch-clamp experiments to determine the effects of H_2_S on cardiovascular control and the potential underlying influence on the excitability of SFO neurons.

## Methods

### Ethics Statement

Male Sprague-Dawley rats (Charles River, QUE, Canada) were used in all experiments. All animal procedures were approved by the Queen’s University Animal Care Committee: Protocol 100821 entitled Central Nervous System Pathways Integrating Cardiovascular and Metabolic Function.

### RT-PCR Analysis

The SFO from three rats were removed. The RNA was immediately isolated and purified using the RNAqueous–Micro Kit (Ambion, Burlington, ON, Canada), and the eluted RNA was treated with DNase (Fermentas, Burlington, ON, Canada). The cDNA was synthesized using the SuperScript II Kit (Invitrogen, Burlington, ON, Canada) using random primers. The cDNA obtained was then subjected to PCR for amplification using primers specific for certain neuropeptides (Table 1). Two microlitres of SFO cDNA was added to 25 µL Qiagen Multiplex Mix, 5 µL of Q solution, 16 µL of sterile H_2_O and 2 µL of the 10 mM primer set, which resulted in a final volume of 50 µL. In order to prevent the formation of misprimed products and primer dimers, we activated the HotStarTaq DNA polymerase (contained in the QIAGEN Multiplex PCR Master Mix) by incubation at 95°C for 15 minutes. The reaction tube was then cycled 35 times through a protocol consisting of incubation at 94°C for 30 s, 60°C for 90 s, 72°C for 90 s, and 72°C for 10 minutes. In order to create a negative, no template control, this protocol was then repeated using sterile H_2_O instead of template cDNA. This reaction was prepared five times, once for each primer set. The final PCR products were run and visualized on electrophoresis gel (containing 2% agarose and RedSafe nucleic acid dye), and were sequenced to confirm their identity (The Centre for Applied Genomics, Toronto, ON, Canada).

**Table 1:** Primer sets used for RT-PCR analysis.

Primer	Accession No.	Direction	Sequence	Product Size (b.p.)
GAPDH	NM_017008.4	Sense	5′-TACCAGGGCTGCCTTCTCT-3′	
		Antisense	5′-CTCGTGGTTCACACCCATC-3′	360
CAT	NM_012571.2	Sense	5′-CTGACTTCTTAGGGCGATGG-3′	
		Antisense	5′-TCACGTTTCCTTTCCACTCC-3′	624
3MST	D50564.1	Sense	5′-ACCACTCTGTGTCGTTGCTG-3′	
		Antisense	5′-TGTCCTTCACAGGGTCTTCC-3′	536
CBS	NP_036654	Sense	5′-AGACACCGACTGGTTTCCAC-3′	
		Antisense	5′-ACAGTCAGTCCAGGGTTTGC-3′	262
CSE	NM_017074	Sense	5′-ACACTTCAGGAATGGGATGG-3′	
		Antisense	5′-TGAGCATGCTGCAGAGTACC-3′	174

### Microinjection techniques

Urethane anesthetized (1.4 g/kg) rats (200–350 g) were fitted with indwelling femoral arterial catheters for the measurement of blood pressure (BP) and heart rate (HR). The animals were then placed in a stereotaxic frame and an incision into the skin of the skull was made. A small burr hole was made such that a microinjection cannula (150 µm tip diameter; Rhodes Medical Instruments) could be advanced into the region of SFO according to the coordinates (Bregma −0.7 mm, ventral 4.5 mm, midline) of Paxinos and Watson [37]. After a minimum 2 minutes baseline BP and HR recording, 5 nmol (0.5 µL of 10 mM) NaHS was microinjected by a pressure-driven 5 µl Hamilton micro-syringe over 10 s into this region and effects on BP and HR were assessed. We also determined the effects of microinjection of varying doses of NaHS into the SFO on BP and HR. In the same animals that received 5 nmol microinjections of NaHS, effects of 1 nmol (0.1 µl of 10 mM NaHS) and 10 nmol (1 µl of 10 mM NaHS) NaHS on BP were assessed while, in a separate group of animals, the effects of 100 pmol (0.1 µl of 1 mM NaHS) and 500 pmol (0.5 µl of 1 mM NaHS) NaHS on BP were determined.

At the end of the experiment, the animal was overdosed with anesthetic, perfused with saline followed by 10% formalin through the left ventricle of the heart, and the brain removed and placed in formalin for a minimum of 24 hours. Using a vibratome, 100 µm sections through the region of SFO were cut, mounted, and cresyl violet stained. The anatomical location of the microinjection site was verified at the light microscopic level, by an observer unaware of the experimental outcome.

Based on histological verification of microinjection cannula placement, animals were assigned to 1 of 3 anatomical groups (SFO, non-SFO, or ventricle). Ventricle sites included both microinjections in which electrode went through the SFO into the underlying 3^rd^ ventricle, as well as deliberate microinjections into the 3^rd^ ventricle (Bregma –0.7 mm, midline 0.7 mm, ventral 4.5 mm). Animals with injection sites that were not wholly confined within any of these regions were excluded from analysis. Vehicle (aCSF) injections were not performed for this study, as our laboratory has previously shown that 0.5 µL vehicle injections into SFO are without effect on BP and HR [29], [33]. Further, as the lowest concentrations of NaHS we microinjected were without effect on BP and HR, they effectively serve the function of vehicle controls. Normalized BP and HR data (calculated by subtracting the mean baseline BP and HR data for 60 s before injection from all data points before and after injection) were obtained for each animal 60 s before the time of microinjection (control period) until 120 s after microinjection. Area under the curve (AUC, area between baseline and each BP and HR response) was calculated for each animal for the 120 s time period immediately following NaHS microinjection. Mean AUC for BP and HR responses for each group were then calculated. A one way analysis of variance (one way ANOVA, followed by Neuman-Keuls post hoc analysis) was used to determine whether BP and HR responses observed in response to NaHS administration were different based on anatomical location (SFO, non-SFO and ventricle) of NaHS microinjection, or the amount (100 pmol, 500 pmol, 1 nmol, 5 mol, and 10 nmol) of NaHS administered.

### SFO neuron preparation

SFO neurons were dissociated as previously described [38]. The rats (125–150 g) were decapitated and their brains were quickly removed and placed in an oxygenated, ice-cold artificial cerebrospinal fluid (aCSF), which contained (in mM): NaCl (124), KCl (2.5), KH_2_PO_4_ (1.24), CaCl_2_ (2.27), MgSO_4_ (1.3), NaHCO_3_ (20), and glucose (10). The SFO was identified using a dissecting microscope, and micro-dissected away from the brain. The isolated SFO was then placed in 5 ml of Hibernate media (Brain Bits, Springfield, IL) containing 10 mg papain (Worthington Biochemical, Lakewood, NJ, USA). It was then incubated for 30 minutes at 31°C. Using hibernate media supplemented with B27 (Invitrogen), the SFO was then rinsed twice and triturated gently three times in order to fully dissociate the cells. The solution was then placed in a centrifuge and spun at 200 g for 8 minutes. The supernatant was then removed, and cells were re-suspended in Neurobasal A media (Invitrogen) containing 100 U/ml penicillin-streptomycin and 0.4 mM L-glutamine (Invitrogen), and supplemented with B27. The newly dissociated neurons were then aliquoted on 35 mm plastic bottom dishes (MatTek, Ashland, MA, USA) and incubated at 37°C in 5% CO_2_ for 2.5–3 hours until the neurons were able to adhere to the bottom of the dish. Approximately 2 ml of Neurobasal A (containing B27) was added to each dish and the cells were returned to the incubator. Dissociated neurons were maintained in culture for at least 24 h prior to being utilised for electrophysiological recordings, and these recordings always took place within 4 days of the dissociation process.

### Electrophysiological techniques

Whole cell patch-clamp recordings were obtained from the SFO neurons using a Multiclamp 700B patch-clamp amplifier (Molecular Devices, Sunnyvale, CA, USA). Spike2 (version 7.05 b) and Signal (version 4.08) software programs (Cambridge Electronics Design, Cambridge, UK) were used to control the stimulation and recording parameters. Using a Cambridge Electronics Design Micro 1401 interface, membrane potential and whole cell current data were digitized at 5 kHz and filtered at 2 kHz. The external recording solution (aCSF) had a pH of 7.2 (adjusted using NaOH), measured 280–300 mOsm, and contained (in mM): NaCl (140), KCl (5), MgCl_2_ (1), CaCl_2_ (2), HEPES (10), mannitol (5), and glucose (5). Electrodes were made from borosilicate glass (World Precision Instruments, Sarasota, FL, USA) using a Flaming Brown micropipette puller (P47, Sutter Instrument Company, Novato, CA, USA) and always had a resistance of 3–6 MΩ. The electrodes were filled with an internal recording solution which measured 280–300 mOsm, had a pH of 7.2 (adjusted using KOH), and contained (in mM): potassium gluconate (125), MgCl_2_•6 H_2_O (2), EGTA (5.5), KCl (10), NaATP (2), HEPES (10), and CaCl_2_ (0.1).

SFO neurons were perfused with aCSF at 37°C at an average rate of 1.5 ml/min. Perfusion set-up utilized a gravity perfusion system and a vacuum pump. Using an MP-225 micromanipulator (Sutter Instrument Co., Novato, CA, USA) the recording electrode was positioned in the bath, and slowly lowered to touch the cell membrane of the targeted neuron. Negative pressure was then applied to allow the formation of a GΩ seal. Whole cell access was obtained by applying a consistent pulse of negative pressure. Voltage clamp protocols were initially run to confirm the presence of voltage gated Na^+^ currents in the cell (thus confirming the cell as a neuron). The recording configuration was then switched to current clamp and a minimum of 200 s baseline recording of the membrane potential was obtained prior to experimental manipulation. If the neurons did not establish a stable baseline for at least 200 s, did not have action potentials greater than 75 mV, or had an input resistance less than 500 MΩ, or did not maintain any of these criteria throughout the entire length of recording, they were excluded from further analysis. NaHS (1 µM–50 mM) was then applied via gravity perfusion. Changes in membrane potential were assessed by comparing the mean membrane potential over 50 s periods before, during, and after NaHS administration. Effects were classified as significant if the mean change observed was greater than 2X the standard deviation (SD) of baseline membrane potential measured in the 50 s period prior to NaHS application. Mean responses were compared between different concentrations of NaHS, and in all cases error values presented represent ± the standard error of the mean (SEM). The distribution of the proportion of neurons responding at each concentration was fitted with a Hill function. In experiments measuring changes in neuronal excitability in response to NaHS application, we followed rheobase protocols previously described in the literature [17], [39]. We first determined the rheobase for each neuron (the minimum injected current required to induce one action potential), and then counted the number of action potentials induced by injections of 2X and 3X rheobase current injection (500 ms steps). We then used 1 s stimulating ramps of linearly increasing current from 0–200 pA and 0–500 pA to induce more than one action potential. Values were compared in both the control and NaHS (1 mM) conditions. Only neurons which fired multiple action potentials with magnitudes greater than 75 mV were included in this analysis.

All statistical analyses were performed using GraphPad Prism 6.01 (La Jolla, CA, USA).

### Chemicals and Peptides

Salts used in preparation of the aCSF and the internal solution, and NaHS were obtained from Sigma (Oakville, ON, Canada).

## Results

### Hydrogen sulfide-producing enzyme mRNA present in SFO tissue

**Figure 1:**
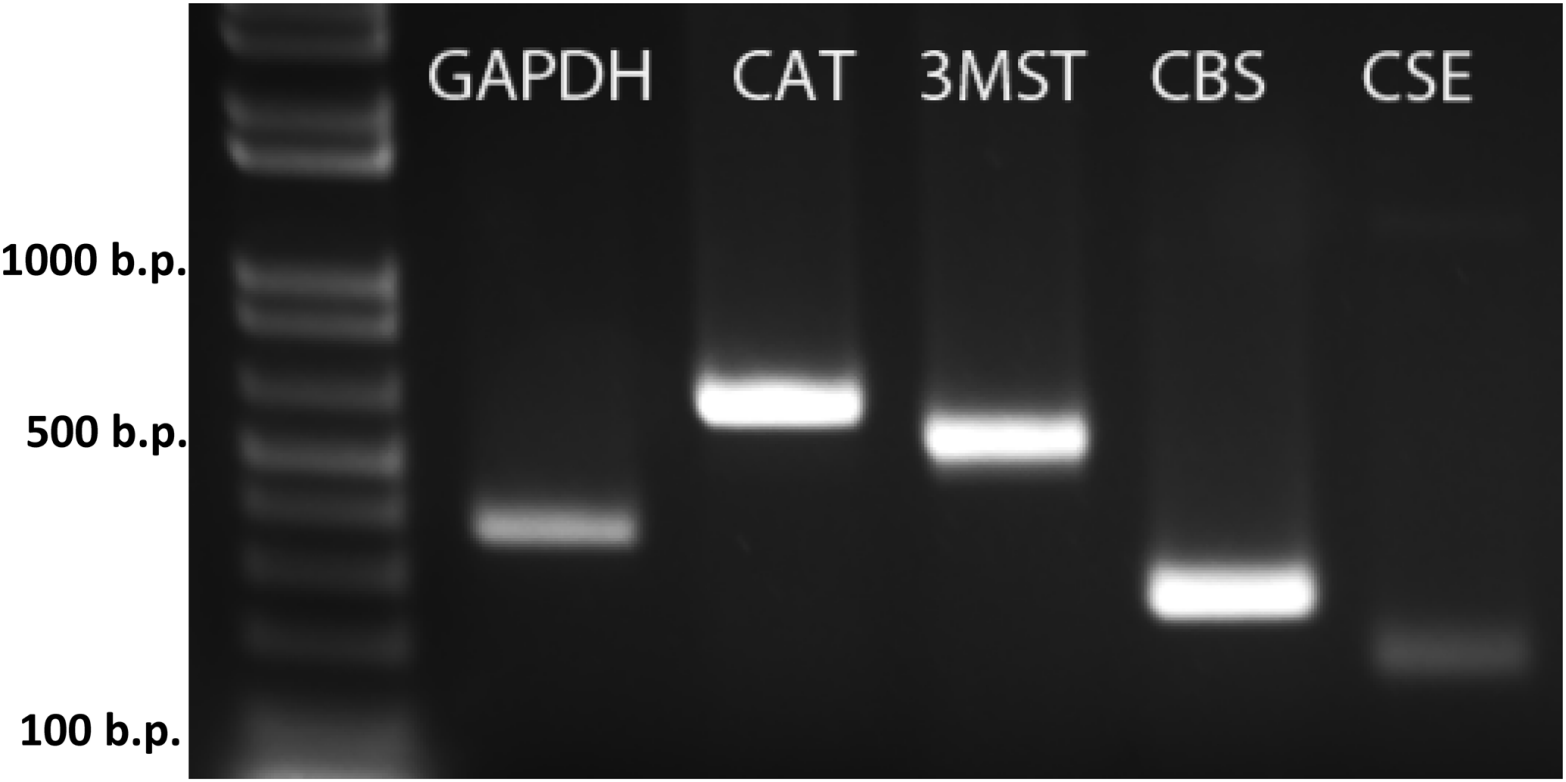
Expression of CAT, 3-MST, CBS, and CSE mRNA in SFO. RT-PCR analysis of SFO shown on an agarose gel with positive bands for the CAT, 3-MST, CBS, and CSE enzymes, as well as a positive control for the housekeeping gene GAPDH.

Our previous microarray analyses suggested that CBS, CSE, 3MST, and CAT were expressed in SFO [36]. We therefore performed RT-PCR on cDNA prepared from mRNA harvested from SFO tissue to confirm that these enzymes were in fact expressed in SFO. The total cDNA underwent PCR amplification using primer sets designed specifically to amplify CBS, CSE, 3MST, and CAT cDNA. As illustrated in the gel shown in Figure 1, the RT-PCR reaction amplified CBS, CSE, 3MST, and CAT. These observations indicate the mRNA responsible for the production of CBS, CSE, 3MST, and CAT is present in SFO, suggesting the SFO as a potential site for both production and also action of H_2_S.

### NaHS microinjection into SFO increases blood pressure

**Figure 2:**
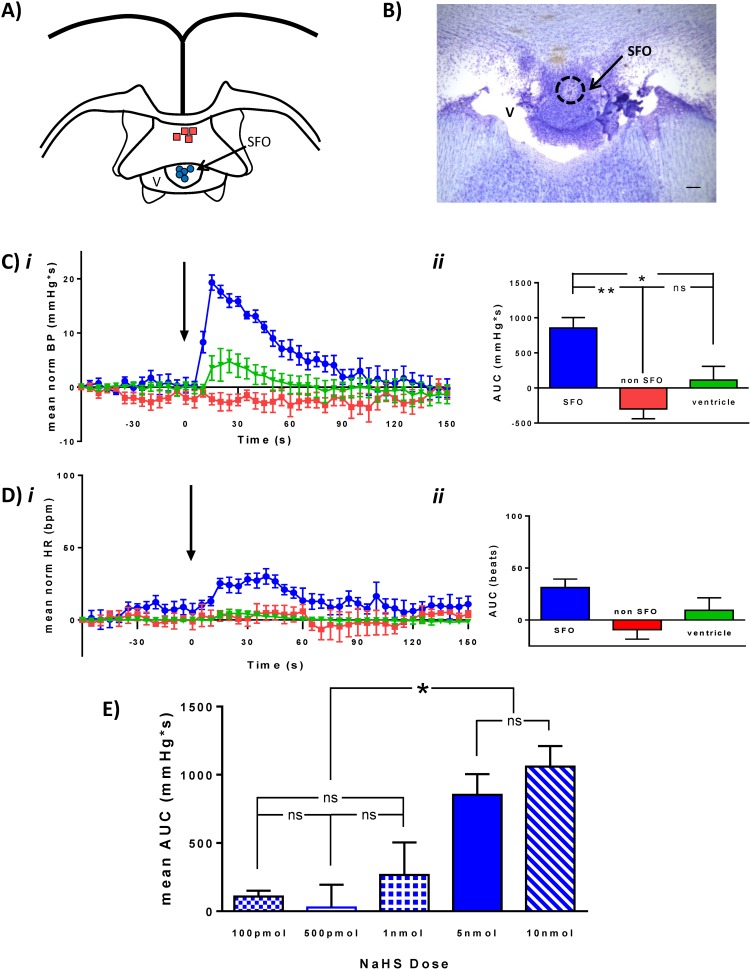
Hydrogen sulfide microinjection into the SFO increases BP. Individual microinjection locations in SFO (Blue circle) and non-SFO sites (red squares) are illustrated in the schematic (A). The photomicrograph (B) shows one of the SFO microinjection sites (scale bar: 100 µm) C) Normalized mean (±SEM) BP trace (*i*) and summary graph (ii) illustrating BP change in response to NaHS (5 nmol) injection (arrow) into SFO (blue, n = 5), non-SFO (red, n = 4), or ventricle (green, n = 5). D) Normalized mean HR trace (*i*) and summary graph (ii) illustrating HR change in response to NaHS (5 nmol) injection (arrow). E) Bar graph illustrating dose-response relationship showing NaHS injections of 100 pmol (boxed), 500 pmol (white), 1 nmol (crossed), 5 nmol (solid), 10 nmol (dashed). Significant differences are indicated by asterisks, *p<0.05. Error bars denote SEM.

A total of 27 animals were used in this study, of which 9 animals had microinjection locations entirely within the SFO (SFO group), 4 animals had microinjection locations outside of the SFO (non-SFO group), and in an additional 5 animals NaHS was microinjected into the lateral ventricle (ventricle group) (Figure 2a, b). The remaining animals (n = 9) were excluded from further analysis as microinjection locations could not be reliably classified into any of the above 3 groups.

Microinjection of 5 nmol (0.5 µL of 10 mM) NaHS into the SFO resulted in increases in BP (mean area under the curve (AUC) = 853.5±150.7 mmHg*s, n = 5) (Figure 2c*i*). These pressor responses were of rapid onset (increase began within 10 s of NaHS administration and peaked approximately 15 s following NaHS administration) and short duration (returning to baseline values within 120 s). These BP effects were found to be site specific (ANOVA, *p<0.01) as NaHS microinjection into non-SFO regions or into the ventricle was without effect (non SFO mean BP AUC = –299.1±140.9 mmHg*s, n = 4; ventricle mean BP AUC = 112.6±93.98 mmHg*s, n = 5) (Figure 2c*ii*). No significant effects on HR were observed (SFO: mean AUC HR = 31.3±8.1 beats (n = 5); non-SFO: mean AUC HR = –9.23±9.1 beats (n = 3); ventricle: mean AUC HR = 9.4±12.0 beats (n = 5); ANOVA, P>0.05) (Figure 2d*i, ii*).

In order to determine whether BP effects elicited in response to NaHS administration into the SFO were dose-related, different volumes of 10 mM and 1 mM NaHS were microinjected into the SFO. The cardiovascular effects observed in response to these different doses were shown to be dose-related (ANOVA p<0.01; see figure 2e). While 10 nmol (1 µL of 10 mM, n = 5) NaHS administration increased BP (mean BP AUC = 1061.0±149.0 mmHg*s), this response was not significantly different from that of 5 nmol (ns: Newman-Keuls post hoc analysis). While not different from each other, the pressor responses to 5 nmol and 10 nmol NaHS were significantly greater than BP responses elicited by 1 nmol (0.1 µL of 10 mM; mean BP AUC = 266.1±237.7 mmHg*s, n = 5; ** Newman-Keuls post hoc analysis vs 10 nmol; * Newman-Keuls post hoc analysis vs 5 nmol), 500 pmol (0.5 µl of 1 mM; mean BP AUC = 9.9±130.0 mmHg*s, n = 5; * Newman-Keuls post hoc analysis) and 100 pmol (0.1 µl of 1 mM; mean BP AUC = 108.0±42.1 mmHg*s (n = 4); * Newman-Keuls post hoc analysis), while BP responses observed at these lower doses (100 pmol, 500 pmol, and 1 nmol) were not different from each other (ns: Newman-Keuls post hoc analysis).

### NaHS depolarizes SFO neurons in a concentration-dependent manner

We used whole cell patch-clamp recordings in current clamp configuration to assess the effects of NaHS on the membrane potential of SFO neurons. Neurons which were included in our analyses were able to fire action potentials greater than 75 mV (either spontaneously or induced by a current pulse of up to 20 pA), had an input resistance greater than 500 MΩ, and exhibited a stable baseline for at least 200 s. Out of 126 neurons tested, 90 neurons met all criteria required for this study. They fired action potentials with mean amplitudes of 99.7±1.6 mV, had a mean input resistance of 1.3±0.2 GΩ, and were recorded from a mean membrane potential of –8.2±1.1 mV.

**Figure 3:**
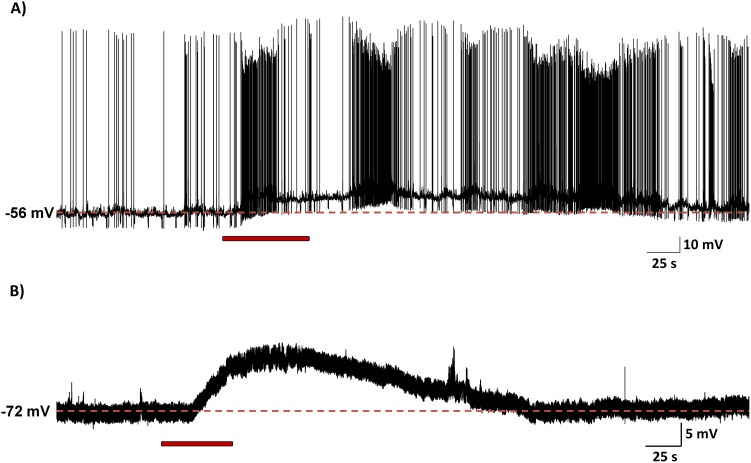
Hydrogen sulfide depolarizes SFO neurons. Current clamp recordings from 2 SFO neurons demonstrating depolarizing effects of 1 mM NaHS (red bars indicate time of application). There was no difference in the depolarizing response observed in neurons that (A) fired spontaneously or (B) cells that were quiescent. In both cases there is a rapid-onset depolarization, followed by a recovery to baseline after return to vehicle aCSF.

In the vast majority of neurons tested, 88 of 90 (97.8%), NaHS (10 µM–50 mM) induced a rapid onset, short lasting depolarization which recovered to baseline shortly after washout of NaHS (mean: 49.8±13.5 s), effects which were normally reproducible in response to a second similar application of NaHS and independent of resting membrane potential or spontaneous activity (Figure 3).

**Figure 4:**
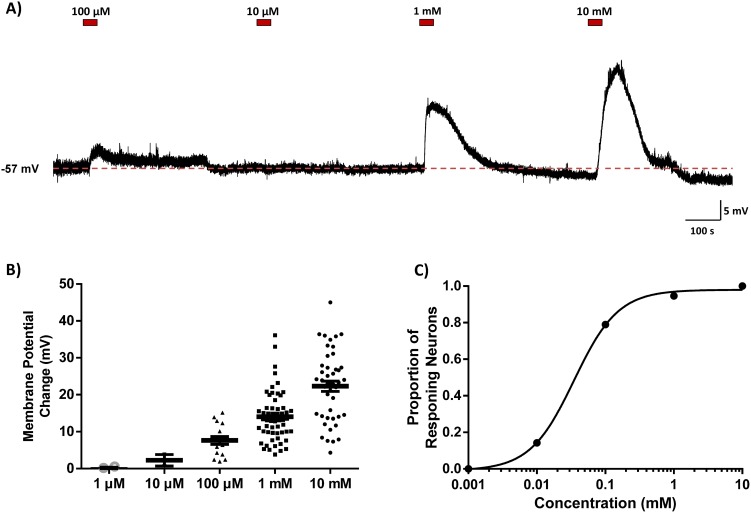
Hydrogen sulfide depolarizes neurons in a concentration-dependent manner. (A) Current clamp recording illustrating the concentration dependent effects of 4 different concentrations of NaHS (red bars indicate time of application) in a single SFO neuron. (B) Scatter plot indicating the membrane potential changes for all neurons recorded. Black bars indicate mean ± SEM. (C) NaHS concentration curve illustrating proportion of responding neurons at each concentration, EC_50_ = 35.6 µM.

NaHS concentrations ranging from 10 µM to 10 mM induced concentration-dependent responses in both the magnitude of the depolarization and the proportion of neurons responsive to NaHS. Bath application of 10 mM NaHS depolarized 32 of 32 (100%) neurons tested (mean: 22.3±1.4 mV); 1 mM NaHS depolarized 53 of 56 (94.6%) neurons tested (mean: 14.0±0.9 mV); 100 µM NaHS depolarized 15 of 19 (78.9%) neurons tested (mean: 7.6±4.2 mV); 10 µM NaHS depolarized 2 of 7 neurons tested (mean: 2.2±1.6 mV); and 1 µM NaHS affected 0 of 3 neurons tested. When recordings were maintained for long periods of time individual neurons were tested with multiple concentrations of NaHS. This resulted in 117 applications of ­ H_2_S in 90 neurons. These data are illustrated in Figure 4a and summarized in Figure 4b. Furthermore, 4 neurons were also tested with a concentration of 50 mM NaHS, and in all cases large depolarizations were observed (mean: 57.9±6.9 mV), although the significant changes in pH and osmolarity in this test solution compared to control aCSF led us to exclude these data from our overall analysis. The proportions of responsive neurons were fitted to a Hill equation, which revealed an EC_50_ of 35.6 µM (Figure 4c).

### NaHS increases the excitability of SFO neurons

**Figure 5:**
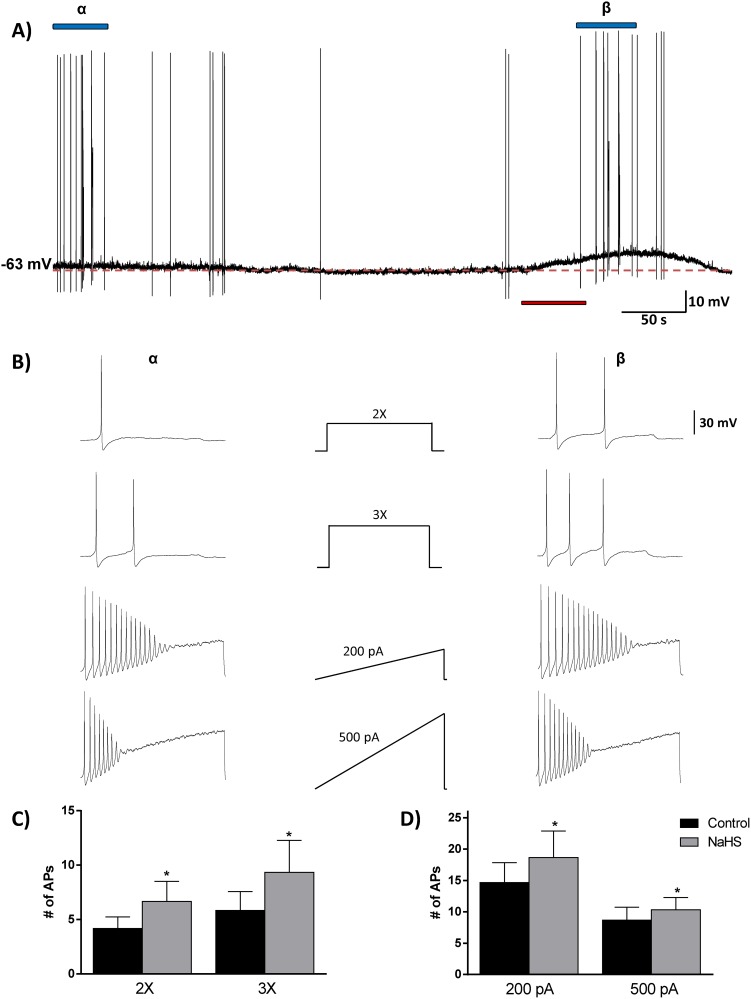
Hydrogen sulfide increases the excitability of SFO neurons. An analysis of rheobase and action potential firing frequency revealed an increase in excitability. (A) Current clamp trace with red bar indicating application of NaHS (1 mM), and blue bars (α and β) indicate periods when current injection protocol was performed. (B) The middle panel shows the current injection protocols for 2X and 3X rheobase steps, and 0–200 and 0–500 pA ramps, while the spike patterns induced are shown for the control (left panel, α) and NaHS (right panel, β) conditions. Bar graphs displaying the mean number of action potentials before (black) and during (grey) NaHS application for the current step (C) and ramp (D) protocols. Note the increase in action potential number in response to NaHS in all 4 cases. *p<0.05, paired t test.

Finally, we examined the effects of NaHS on the excitability of SFO neurons using a simple analysis of rheobase and action potential firing patterns. Rheobase is the minimum amount of current required to induce one action potential, and we used current steps (2X and 3X rheobase) as well as current ramps (0–200 pA/s and 0–500 pA/s) to examine the firing patterns of action potentials before and during application of NaHS (1 mM)(Figure 5a). In all 6 neurons tested, the number of action potentials (APs) induced by the 2X and 3X steps and the 200 pA and 500 pA ramps increased in response to NaHS. The average numbers of APs in response to the 2X current injection in the control phase were 4.2±1.1 AP/500 ms compared to 6.6±1.8 APs/500 ms in the NaHS phase (paired t-test, *p<0.05). The average numbers of APs in response to the 3X current injection in the control phase were 5.8±1.8 AP/500 ms compared to 9.3±2.9 APs/500 ms in the NaHS phase (paired t-test, *p<0.05). The average numbers of APs in response to the 0–200 pA current ramp in the control phase were 14.6±3.2 AP/s compared to 19.0±4.0 APs/500 ms in the NaHS phase (paired t-test, *p<0.05). The average numbers of APs in response to the 0–500 pA current ramp in the control phase were 8.7±2.1 AP/s compared to 10.5±1.8 APs/500 ms in the NaHS phase (paired t-test, *p<0.05). These data are illustrated in figure 5 b*α, β*, and summarized in figure 5c, d.

## Discussion

This study provides the first direct evidence suggesting that H_2_S plays important roles in the SFO, acting as a gasotransmitter to influence cardiovascular function by modulating the excitability of single neurons. Our microinjection experiments, directly applying exogenous NaHS *in vivo* into the SFO of anaesthetised rats, demonstrated site-specific, dose-related effects for this gasotransmitter in controlling cardiovascular function through actions in the SFO. We have used patch-clamp techniques as well as an analysis of rheobase and action potential firing frequency to demonstrate that NaHS depolarizes the majority of SFO neurons, while simultaneously increasing levels of excitability. We have thus uncovered significant H_2_S actions in the SFO, where it acts as an important modulator of cardiovascular function.

We first looked to validate our previous microarray analyses, which suggested the expression of the H_2_S-producing enzymes in SFO [36]. Our RT-PCR analyses confirmed the presence of the mRNA of CBS, CSE, 3MST, and CAT in SFO, suggesting that the machinery for the production of H_2_S is in fact present in SFO. It should be emphasized, however, that detection of the mRNA for these enzymes does not confirm the production or presence of the enzymes, nor does it confirm the action of the enzymes necessary for H_2_S production.

The effects of H_2_S on cardiovascular function have been investigated in a variety of sites in the central nervous system. In association with these studies, we have identified the SFO as a novel site at which H_2_S may act to regulate cardiovascular control systems. Importantly, the observed effects of NaHS on blood pressure were site specific, as microinjection into the ventricle caused no detectable change in blood pressure, as would be expected due to the rapid dilution of NaHS in the ventricle. Furthermore, we saw no apparent baroreflex-mediated decrease in HR in response to the rapid increase in BP, suggesting that H_2_S actions result in a physiologically regulated increase in BP. H_2_S actions in SFO are comparable to the pressor responses elicited in PVN [40], but opposite to the depressor responses elicited in RVLM [18], suggesting anatomically distinct actions for this neural messenger in central cardiovascular control. Apparent conflicts in the literature, where other studies report no effects following H_2_S administration into PVN or RVLM [41], are most likely associated with lower concentrations used in these studies, similar to those at the lower end of our dose-response curve which were also without effect.

We next used whole cell patch-clamp recordings to examine the cellular effects of exogenous NaHS application on the membrane potential of single dissociated SFO neurons. Our current clamp recordings showed an almost universal depolarizing (>97%) action of NaHS. The homogeneity of these effects emphasizes an important role for this gasotransmitter in controlling the membrane potential of neurons in this specific brain area. These effects are similar to those recently reported on PVN neurons (80% responsive, with 96% depolarizing) [19], suggesting potentially common mechanisms of action at the membrane level. We extended these cellular observations using an analysis of rheobase and action potential firing frequency to determine if the NaHS effects on membrane potential were accompanied by increases in “excitability”. We found that NaHS also increased the firing frequency of APs in 100% of the neurons tested. This combination of a depolarized membrane potential and an increase in AP firing frequency results in an overall increase in excitability of SFO neurons in response to NaHS. Stimulation of SFO neurons leads to an increase in BP [42], and it is likely that the uniform increase in excitability and the depolarization of the membrane potential in response to NaHS leads to the observed increase in BP. Our non-SFO microinjection sites were most likely located in the fibres of the hippocampal commissure, and predictably did not cause increases in BP or HR. As such, we believe that our microinjections and bath perfusion of NaHS caused site-specific, effects in SFO. Interestingly, individual SFO neurons have different projection sites and chemical phenotypes (differences our recordings could not distinguish between), and thus, a uniform effect on all neurons located within the SFO may seem physiologically paradoxical. However, the uniformity of responses suggests that H_2_S acts physiologically as a much more specific, locally produced and locally acting messenger. The profound effect on membrane potential may allow for H_2_S produced by one neuron to specifically alter the excitability of their immediate neuronal neighbours, or even themselves, in a rapid and reversible manner, thereby providing a much more precise way of regulating SFO neuron excitability.

In our studies we have used NaHS as an H_2_S donor. Estimates of the amount of free H_2_S in a solution of NaHS in a closed system vary between 6% (personal communication: Dr. J. Wallace) and 18.5% [43] of the original NaHS concentration. Thus, in the open system used in our studies, we would expect a lower percentage (than these closed system estimates) of free H_2_S as a consequence of the even greater dissipation rates. We accounted for this passive loss as best we could by dissolving H_2_S immediately before its use, however 50% is still lost every 5 minutes when dissolved H_2_S is exposed to room air [44]. Taking such considerations into account we would estimate the actual concentrations of H_2_S achieved in this study were likely 3–10% of the NaHS concentrations, suggesting cellular response to H_2_S concentrations between 300 nM and 1 mM. Endogenous active concentrations of H_2_S in the nervous system are even harder to estimate with any accuracy, although these have been suggested to lie between 15 nM and 100 µM [13], [45]–[47], which would require NaHS concentrations of 150 nM–1 mM (assuming 10% values) to mimic this environment. In accordance with such calculations, previous studies in cell expression systems report H_2_S effects on ion channels beginning at concentrations of 1 mM NaHS [48]–[50]. It is however important to interpret such estimates with caution as questions remain as to the mechanism of release of H_2_S from sulfur stores, concentrations in local pools of released H_2_S, and whether free or bound H_2_S functions endogenously to alter cellular function. We therefore used concentrations of NaHS ranging from 100 µM to 10 mM in accordance with previous studies showing reversible effects on membrane potential and ion channel activity [16], [17], [19], [51], concentrations which in our hands elicited effects which were rapidly reversible and repeatable, suggesting that SFO neurons (*in vivo* and *in vitro*) were still viable and healthy after washout of NaHS. In view of these considerations, we believe that effective concentrations of H_2_S achieved in our studies likely fall within high physiological to pharmacological concentrations. When NaHS was dissolved in solution, the change in pH for concentrations ≤1 mM was negligible (<0.1 pH units), while we saw a change of 0.4 pH units to a pH of 7.6 for 10 mM NaHS. These observed changes agree with previously observed values [43]. We bath applied a solution of aCSF adjusted with NaOH to a pH of 7.6 to mimic the pH change which results from dissolving NaHS into solution and saw no effect of this change in pH on membrane potential of SFO neurons.

In conclusion, our studies identify the SFO as an important site of action for H_2_S, and suggest potential implications for the regulation of central cardiovascular pathways. Not only have we demonstrated expression of the genes responsible for the production of H_2_S in SFO, but we also report that microinjection of NaHS directly into SFO caused increases in BP. These observations are in accordance with our patch-clamp analysis of NaHS actions in SFO, demonstrating rapid and reversible depolarizing effects that are associated with increases in excitability in the vast majority of SFO neurons. Future patch-clamp studies will be needed to elucidate the membrane events underlying the increase in excitability of these neurons in response to NaHS, and to describe the specific physiological relevance of these actions in the central regulation of the cardiovascular system.
